# Using peer-ethnography to explore the health and well-being of college students affected by COVID-19

**DOI:** 10.1080/17482631.2023.2261841

**Published:** 2023-09-24

**Authors:** Michelle Teti, Tyler W. Myroniuk, Grace Kirksey, Mariah Pratt, Enid Schatz

**Affiliations:** Department of Public Health, University of Missouri, Columbia, MO, USA

**Keywords:** Adolescent health, college students, COVID-19, interventions, peer ethnography, prevention, qualitative methods

## Abstract

**Purpose:**

COVID-19 continues to infect and affect college-aged youth. We lack information about how students experienced the pandemic day-to-day and what they need for recovery, from their own perspectives. This study employed peer ethnography to explore student’s insights for current and future prevention and care.

**Methods:**

A team of eight students were trained as peer ethnographers to observe and record conversations with their peers in 15-minute increments during the COVID-19 pandemic. Transcripts of 200 conversations were collated and analysed via theme analysis to identify patterns.

**Results:**

Student conversations revealed dichotomous perspectives about COVID-19. Some students prioritized safety, captured via three themes—caution, rethinking routines, and protecting others. Other students struggled to follow prevention guidelines and took risks, also captured by three themes—parties, denial, and misinformation. A third category of themes captured the results of this dichotomy—tense campus relationships and a health leadership vacuum.

**Conclusions:**

Our findings identify specific locations for intervention (e.g., off campus parties) and needed community collaborations (e.g., bars and universities) for COVID-19 and future pandemics. Our findings suggest that overarching approaches, like harm reduction or affirmation (versus shame), are helpful intervention frameworks. Findings also celebrate the value of peer-ethnography, to learn about pandemics and solutions from the ground up.

## Introduction

COVID-19 presented significant health, social, educational, and economic challenges to college students and their campuses, via campus closures, difficult transitions to online learning environments, lost learning opportunities (e.g., study abroad, internships), and decreased student enrolment (Lederer et al., 2020). During the pandemic, students reported a range of mental health challenges like isolation, anxiety, and depression (Birmingham et al., 2021; Browning et al., 2021) as well as housing and food insecurity (American College Health Association, 2020). Vulnerable campus populations—like underrepresented racial/ethnic groups, low-income students, students with disabilities, international students, and/or students who identify as LGBTQ, among others—faced even more hardships (American College Health Association, 2020; Browning et al., 2021; David et al., 2022). Although we know a lot about COVID-19’s impact on students, we know far less about how students experienced the pandemic day-to-day and what they needed and still need for recovery, from their own perspectives. This study employed peer ethnography to highlight student’s insights for prevention and care.

American college and university students have historically been defined as low-risk for health initiatives—often mistakenly categorized as a privileged, resourced, and healthy monolith (Lederer et al., 2020). The COVID-19 pandemic, however, highlighted the diversity of college students and the fallacies of that narrative (Myroniuk et al., 2022). Although many students did not suffer extreme physical health consequences of COVID-19, at the height of the pandemic, college-aged adults represented the highest prevalence of COVID-19 cases and were the least likely to be vaccinated or to report plans to be vaccinated for COVID-19 (Nguyen et al., 2021). This is potentially dangerous for students and broader campus communities (Lennon et al., 2022).

In September, 2022, *Inside Higher Ed* published “COVID goes back to School” about the spike in COVID cases and subsequent limited quarantine spaces on campuses (Inside Higher Ed, 2022). At the time of the writing of this article (December, 2022), warnings of new COVID-19 variants, co-occurring flu outbreaks, and upticks in COVID-19 hospitalizations are frequent (Centers for Disease Control and Prevention (CDC), 2022; New York Times, 2022). Colleges are like “landlocked cruise ships” (Lederer et al., 2020, p. 14). The shared living and learning spaces that define college campuses are ideal grounds for respiratory illness outbreaks like COVID-19 (Fox et al., 2021; Teran et al., 2020; Vang et al., 2021; Wilson et al., 2020), on campus and in college town communities (Lennon et al., 2022).

Despite their actual risk for COVID-19, college students *perceive* themselves at less risk than older adults, and have been less likely to engage in mitigation behaviours (e.g., hand washing, social distancing, and avoiding public or crowded places) that protect themselves and others (Hutchins et al., 2020). Even if students do not experience health complications related to COVID-19, they still acquire, and thus are contagious with, the illness. Whether we continue to grapple with variations of COVID-19 or new respiratory or infectious illnesses, stronger mitigation strategies are needed in shared spaces like college campuses (Lederer et al., 2020; Teti et al., 2022). Despite accumulating evidence for the need to support emerging adults, health-promoting interventions have not sufficiently protected college students against the detrimental effects of the current or future pandemics (Hotez et al., 2021)

Many COVID-19 campaigns focus on individual behaviours—with messaging to mask, maintain distance, or wash hands (Centers for Disease Control and Prevention, 2022). Underlying these campaigns is the assumption that the public will take the disease seriously and act to protect themselves (Tunçgenç et al., 2021)—an assumption that has not been proven true among younger populations. Behaviour change is heavily influenced by or requires the cooperation of others and is a social experience (Drury et al., 2021; Tunçgenç et al., 2021). Moreover, in critical times, such as in a pandemic, people tend to seek out others and their perspectives (Dezecache et al., 2020). In addition, emerging adulthood is a specific development phase, characterized by change, identity exploration, and idea experimentation, especially with others (Arnett, 2000). As college-aged adults strive to achieve independence from their parents, they may be heavily influenced by peer pressure as they make health decisions in communal living and social spaces like college campuses (Hutchins et al., 2020). Thus, research about COVID-19 on campus needs to consider college students’ perspectives and priorities to be useful and translatable to practice.

An underutilized, but effective way to better understand the health needs and experiences of college students in their social context is through participatory research. Participatory research is a form of research where researchers and other stakeholders—including study participants—collaborate on study design, management, and dissemination of results (Viswanathan et al., 2004). The research is oriented towards action and includes benefits for both researchers and participants. This type of work usually requires strong relationships between research teams and community members. Understandably, this became harder during the pandemic and led to decreases in this research design to study aspects of the pandemic (Hall et al., 2021). As a result, we lack the insights of this type of work which often include more ground-up ideas from people who are intended to benefit from the results of the studies being implemented.

To prioritize student perspectives, we implemented a peer ethnography study—a form of participatory qualitative research. In addition, although some ethnographic work has been conducted around COVID-19 prevention activities on college campuses (Barrios et al., 2021; Burnell et al., 2022; Mueller et al., 2021), it is mostly limited to observed numbers of safe and unsafe interactions. Limited conversational data about COVID-19 exists (Drury & Stokoe, 2021). Such conversations can reveal the context of behaviours, however, and target specific areas and situations for intervention (Collins et al., 2022; Drury & Stokoe, 2021). Given the havoc wreaked by COVID to college students, the risk for ongoing and future pandemics in this space, the complex nature of emerging adulthood, and lack of peer-centred and participatory COVID-19 research, we explored COVID-19 via peer ethnographic conversations, to glean more robust and supportive ideas to prevent COVID-19 and future pandemics among college students.

## Methods

### Design

Ethnography is a set of qualitative techniques that aim to study and understand the culture, processes, and patterns of a group of people. It is premised on the idea familiarity and trust with the researcher influences what respondents will disclose and share about their experiences. Peer ethnographic study teams include trained members of the community, who observe and document actions and conversations with their peers. As a result, they glean ideas from participants about helpful interventions (Hackett & Hayre, 2020; Price & Hawkins, 2002). In this case, faculty researchers believed that students’ local and insider knowledge would provide access to and facilitate the collection of accurate data that provided a full picture of the experience of COVID-19 among college students.

The research team recruited students early in the COVID-19 pandemic (Fall, September, 2020) through university public health classes to act as peer ethnographers. The team of eight peers—who varied in gender and race/ethnicity—participated in a small training where they learned about qualitative research and ethnography, observation techniques like notetaking, reflectivity and positionality, and ethics including pandemic safety. The student team was asked not to alter their existing activities and only conduct observations in locations that they were already situated in throughout their day such as classrooms, campus spots, parties, apartment buildings, or dorms (Enria, 2022). This ensured both that students were not taking new risks or exposing themselves in different ways to COVID-19, but also that they were in settings where they “fit” and would not “stand out” as they were locations in which they would normally find themselves. The necessary ethics approvals were obtained from the University of Missouri IRB, Project number 2,028,427.

### Procedures

Students were asked to observe their peers in their surroundings in 15-minute increments. They observed general activities and engagement (or not) with COVID-19 prevention guidelines using an observation sheet. They observed in places they would regularly find themselves, and that were common and popular spaces on campus including main student hubs and meeting halls, dormitories, open park spaces, apartment complexes, and classrooms. In addition to completing an observation sheet, students were also asked to take field notes on conversations that naturally occurred with their friends, classmates, or peers or that they overheard about COVID-19 while engaged in observation activities. They were asked to capture everyday conversations about COVID-19 experiences, opinions, and perspectives in paragraph summaries noting the time, date, place of interaction, speakers, and information shared. The data for this analysis includes transcripts of these conversations.

### Analysis

Transcripts of 200 conversations were collated into one 20-page document. The first three authors, including the study PI (author 1) and two peer ethnographers (authors 3 and 4) conducted thematic analysis (Guest et al., 2012) in four steps: data review, initial coding, specific coding, and data matrices. First, this set of authors reviewed the conversations in full and met to discuss key patterns in the data. They generated a list of ten themes (e.g., prevention behaviours, social activities, relationships, University response) and defined those themes in a codebook. Then the 2^nd^ and 3^rd^ author used the codebook to attach these themes to text in the conversations. After one round of coding, they reviewed the findings and decided to consolidate, delete, or divide themes based on emerging findings, similarities and differences between codes, and depth and breadth of codes. They created a final list of themes and conducted a second round of coding to ensure that they captured all relevant examples and text in those themes. The resulting themes are presented in the results section.

## Results

Student conversations pointed to dichotomous perspectives about COVID-19. On one hand, students prioritized safety, which was captured via three themes—caution, rethinking routines, and protecting others. On the other hand, students struggled to follow masking and other safety directions and took part in risky behaviours, also captured by three themes—parties, denial, and misinformation. A third category of themes captures the results of such a dichotomy—tense relationships and a leadership vacuum. All eight themes are detailed below with conversation notes. Peer-ethnographers are noted as “peers” or “peer ethnographers” below and students who conversed with peers are referred to by their relationship with the peer (e.g., friend) or simply “student.”

### Safety: caution, planning, and protecting others

There were many examples in which students expressed concern about COVID-19 and acted with ***caution***, even if there was no direct evidence of an exposure or a problem. For instance, one peer-ethnographer recounted a conversation where her friend explained that she started feeling sick and worried about COVID-19, even though she had not been contacted by the health department and did not have any obvious or sure exposures. She explained that she called someone “I had very close contact with those few days before I started developing symptoms, figuring we both needed to isolate. They reassured me they were on my side and were completely willing to isolate with me.” Although she wound up testing negative, she described that the person she isolated with helped her manage the “guilt I was feeling about getting sick, even though I regularly wear a mask, social distance, and stay away from places that could compromise [my safety from COVID-19].”

Similarly, another peer recounted a conversation in which a friend discussed testing negative but quarantined with another friend anyway “just to be safe” and not spread the virus via any false positive test results. Several peer-ethnographer conversations addressed groups on campus that went above and beyond to act safely including specific sororities that had both COVID-19 and exposure to COVID-19 reporting lists and systems to deliver food and care supplies to group members who were quarantined.

Students cited examples ***of replanning routines and events*** in which they re-envisioned the ways that they went about “normal” activities that college students partake in during the pandemic. Several peers discussed dating during COVID-19 with students. For example, one peer-ethnographer recounted a conversation with a friend:

She told me she had a date the next day with a girl she had met over [online platform]. Due to the COVID pandemic, she told me they would be ordering take out and going to Peace Park to eat and then converse with masks on. She has been very anxious about the COVID pandemic but is excited to go on a first date in a safe way.

A few other students discussed parts of their social and personal routines with peers, like going to the gym, that they reconsidered in new and safer ways. This included exercising at home given how little masking was enforced at local gyms. Others described ways of altering holiday traditions to feel safer, with peers. For example, one peer-ethnographer recounted this conversation with a student:

I was having a conversation the other day about if and how we might handle Halloween this year. The other student suggested that we could implement some sort of system to hand out candy but to still maintain a safe distance, like the analogy of going to the grocery store but having someone else put your food into the cart for you.

Similarly, another peer-ethnographer discussed with a student how she and her friends managed Thanksgiving by celebrating only with their “quarantine partners” or small social group. She said that this small group of friends “agreed to take great care in spending time with and ensuring we don’t put each other at risk.” Several others also discussed agreements that they made with others to “stay within their bubble throughout the entire pandemic” and about engaging in only safe behaviours (e.g., “fully vaccinated, wear masks everywhere”).

A few conversations centred around ***protecting the vulnerable*** by engaging in safe behaviours to protect others, especially immunocompromised family, friends, and community members. For example, a student who identified as immunocompromised told a peer ethnographer that she had to fight constantly to explain to others that her concerns about COVID-19 were not just her opinions but imperative to her safety. Similarly, another conversant mentioned fear for her immunocompromised friends and yet another lamented a vulnerable student he knew who had COVID-19 and was still suffering from shortness of breath. He told the peer-ethnographer he was worried about this.

Another student discussed with a peer how she felt the burden of taking care of her family members. She said, “my father who is almost 50 years old, has asthma, multiple autoimmune disorders, and heart/lung damage.” She was one of many that shared health problems among family members. Other students mentioned wanting to protect vulnerable populations at their jobs. One peer-ethnographer recounted a conversation—“He had asked where I work and after telling him I work at a respiratory clinic we started talking about people going on oxygen after getting the virus.”

In sum, some students prioritized safety. They were cautious and careful. They were willing and able to rethink their college social events and experiences in new ways that reduced exposure and protected themselves and others. They did their own well-being, but also to protect and with consideration of others who had pre-existing health conditions.

### Risk: parties, denial, and misinformation

On the other hand, many students struggled to stay safe, in part because they were unwilling or unable to make changes to their behaviour. The ***desire to go to parties*** was one area where this tendency emerged. The essence of many conversations was that “guidelines were followed in public just so they could be violated later at parties.” While campus spaces were often safe or partially safe due to rules and regulations set by the institution, the situation was the opposite off campus. Students told peers that this made it challenging for some students to prioritize safety in any location—as they understood that they did not have control over what their classmates did in their free time.

Many conversations were recorded at or about off campus parties. Most hinted that student’s “desire to drink and party outweighs safety concerns.” One peer-ethnographer remarked that her conversations revealed that most students felt that “we are just going to be sent home anyways, so might as well party while we can.” One student described being at a party where “one of the guys just tested positive” and “no one was wearing masks.” Often students talked to each other about the extent of the partying. One recounted to a peer, “one of my friends saw about 20 girls go into the same off-campus apartment.” Another noted,

My friends who live on [part of] campus saw a party of 50–100 people get broken up at their small apartment complex. The party was in a 2-bedroom apartment and the cops were apparently called. When the police showed up, everyone scattered, and few, if any, of the partygoers were wearing masks … Parties have been a regular occurrence this semester, especially since the bars started closing at 10pm.

The bars were also spots of risky behaviour. Many students recounted conversations with peers about crowds of unmasked students in bars despite local masking ordinances, such as this one:

My friend was at a local bar downtown. They had to wear masks as they walked in. They took them off when they got to the table. There were people on an outside patio having a dance party and not social distancing outside and getting in large groups.

The Greek community was also a common site for parties. Several peer-ethnographers recounted hearing conversations like “The girl in the hallway kept exclaiming that she was so excited there were parties starting to happen in Greek town.” One student was part of a conversation in which someone described how a fraternity member “had COVID but it’s okay, he will party with us anyway.” Based on peer conversations with students, off campus parties were a key site for risk behaviour.

A second theme related to risk was ***denial and desperation to be “normal.”*** Many students told peer-ethnographers about their despair at the way COVID-19 had uprooted college life and a desperation for things to be back to normal. This partially explained students’ risky behaviours. For instance, one peer-ethnographer recounted this conversation:

My friend attended a party. No masks were worn at this party and there were approximately 30 people. Most people attending the party already had [had] COVID. People seemed unbothered as everyone had already had it and people were immune. It was outside by the fire, and everyone was drinking and hanging out. Nobody seemed to care about everything going on and acted as everything was normal.

Another peer described a conversation about study rooms in the dorms, saying, “It was evening time and people were in study rooms working on homework with others. People didn’t seem to be wearing any masks and it felt like everything was back to normal.”

Relatedly, other students talked with peer-ethnographers about COVID-19 denial. One said, “my friend said one of their roommates told her she ‘does not even think COVID is real’ and was trying to peer-pressure her to go to a party.”

The question of “what was true” permeated conversations with peers. Students said they heard and shared ***misguided information*** creating confusion about how students should protect themselves from COVID-19. Many peer-ethnographers talked to other students about incorrect mask use, such as masks partially down or not covering faces. A significant area of confusion was around if someone still needed to mask or social distance if they were vaccinated. One peer noted:

I hear conversations of people saying they are vaccinated in a way of justifying them doing something that would normally be against COVID guidelines. This is mostly around justification of going to a party or having large gatherings with unmasked people. Someone might say something like “I don’t know if we should go [to this place because of] COVID and everything” and someone will respond, “It’s okay, we’re vaccinated.”

There were also questions about vaccination and the side effects. It was common for peers to recount issues about side effects from their conversations with students. One student mentioned she had a friend in a sorority who feared the vaccine and said, “her reasoning was because the people in the sorority had heard the vaccine would make you infertile, so none of them were going to get it.”

Lastly, there was much confusion about how much protection, or not, immunocompromised students needed. This student summarized the issue with this example conversation that she discussed with a peer-ethnographer:

This guy from my hometown texted me and asked me when homecoming was. I’ve told him multiple times that I am immunocompromised, and he grew up with me so he KNOWS. After I told him when homecoming was, he said “Okay if I’m up there are you open to going out? I get your opinions on the ‘rona’ thing so it’s no worries either way.” I replied, “It’s not an opinion, I could die.” He got upset that I wouldn’t see him because he has the antibodies now so he “can’t hurt me.”

In sum, based on peer-ethnographers’ conversations, another cadre of students faced difficulties to following masking and social distancing guidelines. They prioritized social events, expressed denial of COVID-19, and admitted being confused about the facts related to transmission of and the needs to protect oneself from COVID-19.

### Safety versus risk: consequences

College is a social experience and COVID-19 is spread socially. Varying COVID-19 prevention stances and approaches came into direct conflict within students’ relationships with each other. Relatedly, while there is the potential for some self-sorting by values and belief statuses, even college students who chose friends and roommates based on other similarities, confronted new differences regarding COVID-19. Students who fit with those who focused on safety had to regularly interact with those who were more comfortable with risk (e.g., roommates, classmates), creating ***relational tensions***. According to peer conversations, a major source of tension during COVID-19 was managing intimate partner and roommate relationships. Given how easily COVID-19 spreads, roommates were forced to work through COVID-19 together. One student told a peer-ethnographer that “COVID-19 was like an STD” because of how everyone’s risk was connected. In several examples peer-ethnographers overhead roommate arguments.

I overheard a major fight between roommates when I was over at a friend’s apartment. One roommate had gone to a crowded house party the night before where no one was wearing masks or maintaining social distancing … . and the other two roommates had confronted her about this (one of the two roommates is immunocompromised herself, and the other has frequent contact with their grandparents). The roommate who went to the party became very defensive and said “If you two get COVID-19, that’s your problem. I’m going to live my life how I want to. I don’t care what you think,”

Similarly, another peer-ethnographer recounted hearing this conversation between a young woman and her boyfriend about socializing:

My neighbor and her boyfriend were arguing audibly through our walls about seeing each other’s friends. She was angry because he wanted them to hang out with his friends who had not been tested for COVID and she hung out with them all, but he would not hang out with her friends even though they had tested negative for COVID recently. They proceeded to yell until she stormed out and left the building.

In other examples, peer-ethnographers discussed their own experiences. One explained that she had to ask her roommate to wear a mask in shared spaces of their apartment because the roommate had just visited a large festival like camping event. Her roommate however, “didn’t see the need to, even though she had been around a group of 50+ strangers for three days … and admitted she had been to a house party the previous week and one of the guys just tested positive.” Another peer-ethnographer described a situation in which three friends made an agreement about not going to bars, yet later, “one of the three admitted to frequenting bars without telling us.”

While interpersonal tensions were common, another result of the disconnect between types of student behaviour, was the ***leadership vacuum*** related to COVID-19 on campus that many students experienced. Conversations revealed that students felt like they lacked clear leadership on COVID-19; they did not trust the university’s efforts at prevention or protection. They discussed hearing about and their own confusing quarantine experiences, where they were not sure how they would be fed in quarantine or how they would know when quarantine was over. Some students belonged to organizations on campus and looked to them for guidance, but many of these organizations did not fill the gap well. One peer-ethnographer wrote about how students felt about Greek organizations, “Some of the houses do not enforce mask-wearing, others are strict. There is not someone that is overseeing the rules [across Greek Life].” Others looked to their professors, but classroom behaviour was also inconsistent. Students reported enforced masking in some classes but not others and professors who masked correctly and others who “pulled their masks down while teaching.”

Resident Assistants (RA) were also not consistent sources of information or actions, either. One peer-ethnographer wrote:

I was talking to a friend who works as an RA. He was talking to me about how the RAs are put in an uncomfortable position because they can’t do regular room checks, but they could also be fired if they have residents violating the rules and they don’t report it.

Others knew about outbreaks on their floor but were not formally notified by the university or the RA. Other RAs tried to go above and beyond to help, which was stressful. One peer-ethnographer talked to an RA and noted that he said:

He made sure to pick up an RA shift for Halloween night because he knew that would also be a crazy weekend with a lot of violations, and he didn’t trust many of the RAs to do their job and report people on days like this.

Support did not come from outside of the university either. Many peer-ethnographers recounted conversations with students about COVID-19 communication with family members. One said he went to his father for masking guidance, but said his father did not engage, saying he doesn’t wear a mask in town because he doesn’t want people to “judge him.” Another said their brother told them that “most people around me, we have much more problems than a lousy disease. There’s SO many worse things that could kill me. You must figure out what’s best for yourself and stick to that.” Yet another said their grandmother was high risk but told her that prevention was pointless because “the world was ending anyway.”

Conversations told a story of students having to navigate opposing viewpoints about COVID-19 and why and how to stay safe. Students’ actions occurred at both ends of the spectrum, some enacting extreme caution while others lived in denial, making it challenging for students to make choices about their own behaviour, while also considering how it might affect others. In this context, students looked for leadership on how to behave and what was “true,” but instead experienced a vacuum in leadership and confusion about where to turn for help. In addition, on a more relational level, students were forced to manage many difficult conversations and relationship tensions without help.

## Discussion

Data about the effect of COVID-19 on students exists, but far less guidance is available about what is needed to support students in this and future epidemics, especially from a participatory research framework and/or students’ perspectives (Daly Lynn et al., 2022). In this study, conversations among or overheard by peer-ethnographers revealed specific and contextual points of concerns where health information and behaviour interventions are needed such as vaccines, off-campus events, conflict resolution, and immunocompromised students. It is possible that using peers as informal interviewers improved the reach of our study and the comfort—and therefore honesty—of participants (Devotta et al., 2016).

Research describes how students made the decision to get vaccinated for COVID-19 (Varol et al., 2022). To our knowledge, existing research does not explore specifically how college students made decisions about their risk-taking during the pandemic regarding attending parties or large gatherings, and how this relates to vaccine status. It is possible that these decisions match factors considered in other common adolescent risk behaviour situations—like smoking, drinking or safe sex—and are aligned with factors of common health models used to understand such risks. As cited by the Health Belief Model (Downing-Matibag & Geisinger, 2009) or Social Cognitive Theory (Dos Santos, 2020), behaviour is complicated and depends on beliefs, risk perceptions, self-efficacy, peer influence and social norms and pressures, among other factors. For example, messaging about how vaccines can play a part in bringing a campus “back to normal” may be helpful for students who indicate a strong desire to take back their expected college experience.

Our findings support others that indicate that off campus behaviours (e.g., parties, large gatherings) were far more problematic than on campus behaviours (e.g., classes) during the active COVID-19 pandemic (Kianersi et al., 2021). Although few college campuses were able to truly hold students accountable for unsafe actions like failure to mask or social distance (Teti et al., 2022), off-campus activities were described by students in our study as essentially rule-less when it came to virus prevention. Many universities, including the site of this study, only focused on on-campus activities because these were in their domain or control. This makes logistical sense but fails as a public health strategy. Baseline awareness—that campuses and their adjacent settings need to collaborate—is necessary.

Our findings suggest that colleges partner with campus (e.g., Greek) and community (bars, apartments) to discuss problems and potential solutions, create signage, and develop ways for students to report problematic behaviour. Placing signs and instructions on campus are not sufficient to stop the spread of the virus. In addition to information, students craving social activities need outlets such as socially distanced or online events. Our results suggest that if students are going to party, harm reduction frameworks (e.g., how to stay safe while partying) versus binary messaging (do not party during COVID-19) are more helpful for off-campus points of risk behaviours. This is especially important given the role that alcohol and parties play in college social activities (Brown & Murphy, 2020).

Second, a particular area of confusion noted by peer-ethnographer conversations was about the COVID-19 vaccine, including side effects and safety concerns. This supports other research indicating that young people report low vaccine uptake (Nguyen et al., 2021). Colleges could focus on these fears with materials that dispel vaccine myths and tailor them appropriately to specific risk groups. For example, in our study, women expressed concerns about the vaccine’s effect on pregnancy. Materials and educational sessions could target specific groups (e.g., sororities, women’s spaces on campus) with vaccine facts—and help information recipients learn how to talk about the vaccine with others.

On that note, our findings revealed dichotomous approaches to COVID-19 among students whose conversations were captured in this study. Of course, there is a middle ground, but we believe it is noteworthy that our participants reported conversations mostly at the two ends of the spectrum. On one hand, students were struggling or worrying about staying safe; on the other, they were mired in denial and misinformation. What this does suggest is that there are many opposing perspectives to how to manage COVID-10 on campuses. College students do not always choose their roommates and in our study, many conversations described conflict between roommates, friends, and partners, over clashing COVID-19 perspectives. This caused stress for many respondents, especially given that unlike other health risks (e.g., lack of sleep, too much drinking) are less communal than COVID-19, in which one student’s behaviour can directly harm another’s.

Interventions could support college students in talking to others about COVID-19 and other infectious illnesses and help students talk and work together to ensure both parties feel safe. Such interventions can help students describe their motivations to stay safe to others, manage conflict, and negotiate solutions. Conflict resolution interventions for college students are rare but may help students address many different elements of difficult social and health interaction. For example, a recent study of college students indicated that they wanted to explore the social and communication aspects of sexual activity to engage in safe sex (Astle et al., 2021). Similarly, interventions like Green Dot that encourage students to talk to and protect each regarding difficult issues like violence (Coker et al., 2016), may also provide a helpful model for COVID-19.

Nearly 6% of college students report chronic illness such as cancer, diabetes, or auto-immune disorders (American College Health Association, 2018). In our study, several participants described the challenges of having a compromised immune system amid the pandemic and of negotiating safety on campus. This was supported by a small amount of other research among youth and adults with chronic conditions (Saqib et al., 2020; Serlachius et al., 2020). An additional intervention point could include educating the general student population about immunocompromised students’ needs, the importance for such students to follow guidelines more adamantly or ask for safety and focus on virtual or other support systems for these students specifically.

Our findings also highlight that some students are doing many things correctly to protect themselves and others. While fear and shaming has had little success in promoting behaviour (Fairchild et al., 2015), positive reinforcement and peer modelling is a promising health promotion tool (Epton et al., 2015). Colleges may be able to utilize such students as role models for others or keep these students motivated to engage in safe behaviours by rewarding students or student groups for creating alternative events, for example. Some students in our examples were willing to recreate new routines and could pave the way for others to do the same.

Lastly, our findings pointed out the dire consequences of lack of leadership or guiding voices during a crisis like COVID-19. Students reported looking for support from professors, RAs, their families and even university and community health administrators. No clear guiding voices emerged. This left the students scared, confused and sometimes angry. It is important for colleges to follow the guidelines for any emergency in this situation including a clear point of command, information, and guide for next steps (CDC, 2022). Students want to understand the rules, the accountability structures, and how to voice their questions or concerns when set processes are not working. Clear information and places to go for help would improve the COVID-19 response for young people.

There are limitations to our work. Conversations were limited to those enacted or heard by the peer-ethnographers and occurred in spaces in which our peer-ethnographers were situated. Given that all the peer-ethnographers were recruited from public health courses, they may have been more likely to hear conversations and be situated in spaces that promoted public health perspectives on the pandemics. Thus, we did not capture the breadth of all conversations on campus. Peer-ethnographers were asked *not* to add places or locations to their daily routine for this project to protect them from COVID-19 risk beyond what they would normally be exposed to. This also then restricted peer-ethnographers’ spaces and places of observation and the conversations to which they were exposed. Like any qualitative research, then, our results are not generalizable to all on our campus, or to other different kinds of universities (e.g., private, urban). In addition, a structured interview guide may have collected more specific and consistent COVID-19 information.

Our findings suggest specific areas for attention (e.g., off campus parties) and needed community collaborations (e.g., bars and universities) for this and future pandemics. They point to overarching approaches, like harm reduction or affirmation (versus shame), as helpful frameworks for interventions. They also celebrate the value of community-based approaches, such as peer-ethnography, to learn about pandemics from the ground up. While filtered through the peer-ethnographers, the conversations recorded were ones that students were having over the course of the pandemic, rather than being prompted by researchers. Such methods are vital to knowing what pandemics look like on the ground and where resources are needed. This is especially important given the wide range of behaviours and perspectives involved in COVID-19, and the varied experiences of college students over the course of the pandemic.

## Geolocation information

Midwest, United States
